# Estrogen Regulates MAPK-Related Genes through Genomic and Nongenomic Interactions between IGF-I Receptor Tyrosine Kinase and Estrogen Receptor-Alpha Signaling Pathways in Human Uterine Leiomyoma Cells

**DOI:** 10.1155/2012/204236

**Published:** 2012-10-09

**Authors:** Linda Yu, Alicia B. Moore, Lysandra Castro, Xiaohua Gao, Hoang-Long C. Huynh, Michelle Klippel, Norris D. Flagler, Yi Lu, Grace E. Kissling, Darlene Dixon

**Affiliations:** ^1^Molecular Pathogenesis Group, National Toxicology Program (NTP) Laboratory, NTP, Department of Health and Human Services (DHHS), National Institute of Environmental Health Sciences (NIEHS), National Institutes of Health (NIH), Research Triangle Park, NC 27709, USA; ^2^Cellular and Molecular Pathology Branch, NTP, Department of Health and Human Services (DHHS), National Institute of Environmental Health Sciences (NIEHS), National Institutes of Health (NIH), Research Triangle Park, NC 27709, USA; ^3^Biostatistics Branch, Department of Health and Human Services (DHHS), National Institute of Environmental Health Sciences (NIEHS), National Institutes of Health (NIH), Research Triangle Park, NC 27709, USA

## Abstract

Estrogen and growth factors play a major role in uterine leiomyoma (UtLM) growth possibly through interactions of receptor tyrosine kinases (RTKs) and estrogen receptor-alpha (ER*α*) signaling. We determined the genomic and nongenomic effects of 17*β*-estradiol (E_2_) on IGF-IR/MAPKp44/42 signaling and gene expression in human UtLM cells with intact or silenced IGF-IR. Analysis by RT^2^ Profiler PCR-array showed genes involved in IGF-IR/MAPK signaling were upregulated in UtLM cells by E_2_ including cyclin D kinases, MAPKs, and MAPK kinases; RTK signaling mediator, GRB2; transcriptional factors ELK1 and E2F1; CCNB2 involved in cell cycle progression, proliferation, and survival; and COL1A1 associated with collagen synthesis. Silencing (si)IGF-IR attenuated the above effects and resulted in upregulation of different genes, such as transcriptional factor ETS2; the tyrosine kinase receptor, EGFR; and DLK1 involved in fibrosis. E_2_ rapidly activated IGF-IR/MAPKp44/42 signaling nongenomically and induced phosphorylation of ER*α* at ser118 in cells with a functional IGF-IR versus those without. E_2_ also upregulated IGF-I gene and protein expression through a prolonged genomic event. These results suggest a pivotal role of IGF-IR and possibly other RTKs in mediating genomic and nongenomic hormone receptor interactions and signaling in fibroids and provide novel genes and targets for future intervention and prevention strategies.

## 1. Introduction

Although the exact etiology of uterine leiomyomas (fibroids) is unknown, the fact that they develop during the reproductive years and regress after menopause indicates that they are hormonally regulated [1–3]. The important role of estrogen in the promotion of uterine leiomyoma growth has been well supported through clinical and biological studies [4–6]. However, the overexpression of growth factors and their receptors, such as the type I insulin-like growth factor (IGF-I) and IGF-I receptor (IGF-IR), shows that sex steroids are not the only modulators of leiomyoma cell proliferation and exuberant extracellular matrix formation observed in many fibroids [2, 7–9]. Studies have revealed that IGF-I expression is most abundant in leiomyomas during the proliferative phase of the menstrual cycle [10, 11]. The expression of IGF-I mRNA increases in leiomyomas, and estrogen receptor alpha (ER*α*) mRNA is positively correlated with IGF-I mRNA levels, which implies as we and others have shown that estrogen upregulates the gene encoding IGF-I through ER*α* in leiomyoma tissue and cells [10–13]. 

The accumulated data from extensive studies of breast cancer, another disease that is often hormonally regulated, have shown that the interactions of estrogen/ER*α* with IGF-IR/MAPK signaling can occur at different molecular levels [14, 15]. It is known that 17*β*-estradiol (E_2_) primarily acts through cognate nuclear ER*α* leading to regulation of gene expression, which has traditionally been defined as genomic estrogen activity. It has also been reported that many of the E_2_-responsive genes are key signaling molecules that participate in IGF-IR signaling [16]. Alternatively, a cell membrane-associated form of ER*α* has been reported to couple with and activate IGF-IR through phosphorylated Shc [17, 18], thereby triggering rapid nongenomic effects through transactivation of the IGF-IR. More recently, our and other studies have shown that E_2_ and environmental phytoestrogens (genistein) can induce E_2_-dependent signals prompting major biological responses such as gene expression and human uterine leiomyoma (UtLM) cell proliferation [12, 13, 19]. In addition, E_2_ is able to upregulate IGF-I gene expression [12, 16, 20] and increases IGF-I synthesis, which leads to activation of IGF-IR*β* and MAPKp44/42 [19, 21]. Increased activated MAPKp44/42 with enhanced phosphorylation of ER*α*-phospho-ser118 has been observed in uterine leiomyomas, but not in uterine smooth muscle tissue [22]. 

 All of these studies suggest that the effect of growth promoter IGF-I and its receptor IGF-IR and downstream target MAPK-related genes may be regulated by estrogen at genomic and nongenomic levels, and the interaction of ER*α* and IGF-IR/MAPKp44/42 may play a pivotal role in fibroid tumorigenesis, but the exact mechanism(s) of how this all occurs is unknown. There are several questions that need to be addressed such as (1) how does estrogen regulate MAPK related gene expression through IGF-I and the IGF-IR/MAPKp44/42 pathway; (2) what is the role of E_2_ in the activation of IGF-IR leading to MAPKp44/42 phosphorylation of ER*α* at the serine 118 site; (3) which specific mediators of the signaling pathways are involved in the interactions of IGF-I/IGF-IR and ER*α* in uterine leiomyomas. In this study, we explored the regulatory effects of E_2_ on intermediates of the IGF-IR/MAPK signaling cascade and related gene expression in UtLM cells and identified new genomic mediators involved in fibroid growth and development by genomic array profiling. We also determined if the effects of E_2_ were through genomic and nongenomic interactions of IGF-I, IGF-IR, ER*α*ser118, and MAPKp44/42 by silencing IGF-IR followed by western blotting, immunoprecipitation, and immunofluorescence microscopy. To our knowledge, this is the first study in human leiomyoma research focusing on determining the profile of E_2_-mediated IGF-IR/MAPK-related genes and exploring the genomic and nongenomic interactions of ER*α*/IGF-IR/MAPKp44/42 pathways. 

## 2. Material and Methods

### 2.1. Cell and Cell Culture

UtLM cells (GM10964) were purchased from Coriell Institute for Medical Research (Camden, NJ, USA) and maintained in MEM (Gibco Life Technologies, Grand Island, NY, USA) with supplements at 37°C, with 95% humidity, and 5% carbon dioxide as previously described [9]. 

### 2.2. siIGF-IR and Real-Time Profiler PCR Array

The RT^2^ Profiler PCR Array System (MAP Kinase Signaling Pathway PCR Array, PAHS-061) from SABiosciences (Frederick, MD, USA) was used to analyze gene expression related to the MAPK signaling pathway in UtLM cells in response to E_2_ (Sigma, St. Louis, MO, USA) treatment, with and without IGF-IR silencing (siIGF-IR). The transfection of siIGF-IR oligo targeting human IGF-IR gene (5′ to 3′ CGUCUUCCAUAGAAAGAGAtt) and a control scrambled siRNA (siScr) with a nonsense sequence designed to have no significant sequence similarity to mouse, rat, or human transcript sequences (Ambion, Foster City, CA, USA) into cells was done using Lipofectamine (Invitrogen, Carlsbad, CA, USA) as a transfection agent following the manufacturer's protocol. UtLM cells were incubated in serum-free medium containing 0.001% of vehicle (ethanol) for 24 h after siIGF-IR, then treated with E_2_ (10^−8^ M), and harvested at 24 h using Trizol Reagent (Invitrogen). Total cellular RNA was extracted from the cells using Qiagen RNeasy Mini Kit (SABiosciences) followed by the RT^2^ First Strand C-03 Kit (SABiosciences) to remove any residual contamination from the RNA samples with 2 *μ*g purified RNA per treatment condition. The template combined with the RT^2^ SYBR green/ROX qPCR mix (25 *μ*L/well) was loaded into a 96-well array plate coated with 84 predispensed MAPK-related gene-specific primer sets (SABiosciences) with each treatment condition per plate and processed on a TaqMan ABI Prism 7900 Sequence Detector System (Applied Biosystems, Foster City, CA, USA) according to the RT^2^ Profiler PCR Array (SABiosciences) manufacturer's protocol. The data analysis was based on the ΔΔC_*t*_ method with normalization to GAPDH and HPRT (SABiosciences), (http://www.sabiosciences.com/pcrarraydataanalysis.php).

### 2.3. Real-Time PCR

Real-time (RT) PCR was performed to detect estrogen responsive gene, IGF-I mRNA expression levels following E_2_ treatment at 0 min, 10 min, 60 min, 24 h, and 48 h in UtLM cells. Starvation of cells with serum-free medium was the same as described for RT^2^ Profiler PCR Array and occurred 24 h prior to E_2_ (10^−8^ M) treatment. Cells were harvested with Trizol Reagent, and total cellular RNA was extracted from the cells using a Trizol Plus RNA Purification Kit (Qiagen, Valencia, CA, USA). One microgram total RNA was used to prepare cDNA and reverse-transcribed with Superscript II (Invitrogen). RT-PCR was performed using IGF-I and GAPDH primers [19] with Applied Biosystems Power SYBR Green PCR Mix on an AB cycler. The results were expressed as fold changes compared to untreated groups and normalized with GAPDH.

### 2.4. Immunofluorescence Staining (Confocal Microscopy)

Immunofluorescence staining was performed to detect IGF-I peptide expression at 0 min, 10 min, 60 min, 24 h, and 48 h, and phospho-ER*α*ser118 and phospho-MAPKp44/42 colocalization at 0 min, 10 min, and 60 min in UtLM cells following E_2_ treatment. The cells were starved the same as described for the real-time RT^2^ Profiler PCR Array for 24 h prior to E_2_ treatment at 10^−8^ M. The cells were fixed with 4% paraformaldehyde (Electron Microscopy Sciences, Hatfield, PA, USA), permeabilized with 0.2% Triton X-100 (Sigma), and blocked with 5% BSA (Sigma) and 0.1% gelatin (Sigma) in PBS. The cells were incubated overnight with primary IGF-I goat polyclonal antibody (sc-1422, 1 : 200 dilution; Santa Cruz Biotechnology, Santa Cruz, CA, USA), phospho-ER*α*ser118 mouse monoclonal antibody (number 2511, 1 : 50 dilution; Cell Signaling, Danvers, MA) and phosho-MAPKp44/42 rabbit monoclonal antibody (number 9101, 1 : 100 dilution, Cell Signaling). Alexa Fluor (Invitrogen) 488 goat anti-rabbit for phospho-MAPKp44/42, Alexa Fluor (Invitrogen) 594 donkey anti-mouse for phospho-ER*α*ser118, and 594 donkey anti-goat for IGF-I (1 : 3000 dilution) were used as secondary antibodies and DAPI (Invitrogen) for nuclear staining. Normal rabbit, normal mouse, or normal goat serum (Jackson Immunoresearch, West Grove, PA, USA) served as negative controls. Confocal images were taken on a Zeiss LSM510-UV meta (Carl Zeiss Inc, Oberkochen, Germany) using a C-Apochromat 40x/1.2 *∞*/0.14–0.19 Korr UV-VIS-IR objective. The 488 nm laser line from a Krypton/Argon laser was used for excitation of the Alexa 488. A 505–550 nm bandpass emission filter was used to collect this image with a pinhole setting of 1.09 airy unit. For the second channel, the 543 nm laser line from a Helium Neon laser was used for excitation of the Alexa 594. A 560 nm longpass emission filter was used to collect the images with a pinhole setting of 1 airy unit.

### 2.5. Western Blot Analysis

Western blot analysis was performed using a standard procedure as described previously [9] to assess rapid effects of E_2_ on target protein expression in UtLM cells. The siIGF-IR and siScr transfection procedure were the same as described in siIGF-IR and real-time RT^2^ Profiler PCR Array. The serum-starved cells were treated with E_2_ (10^−8^ M) at 0, 10, and 60 min. The protein was extracted with a lysis buffer as previously described [9]. Primary antibodies used for the western blotting were as follows: rabbit polyclonal anti-phospho-IGF-1R*β*Tyr1131/IR-*β*Tyr1146 (number 3021, Cell Signaling), rabbit polyclonal anti-IGF-IR*β* (sc-713, Santa Cruz Biotechnology), rabbit polyclonal anti-phospho-Shc (number 2434, Cell Signaling), rabbit polyclonal anti-Shc (sc-288, Santa Cruz), rabbit polyclonal anti-ER*α* (sc-7207, Santa Cruz), mouse monoclonal anti-phospho-ER*α*Ser118 (number 2511, Cell Signaling), rabbit polyclonal anti-MAPKp44/42, and rabbit polyclonal anti-phospho-MAPKp44/42 (number 9201 and number 9101, Cell Signaling). The rabbit anti-ER*α*, rabbit anti-IGF-IR*β*, and rabbit anti-Shc antibodies used in the western blotting studies were used for immunoprecipitation samples. Primary antibodies were detected with horseradish peroxidase-conjugated secondary anti-mouse or anti-rabbit antibodies (GE Healthcare, Buchinghamshire, UK).

### 2.6. Immunoprecipitation

A Seize Primary Immunoprecipitation Kit (Pierce Biotechnology, Rockford, IL, USA) was used to detect the association of ER*α*, IGF-IR*β*, and Shc in the cells treated with E_2_. The kit was used because IgG of anti-ER*α*, which was used to pull down Shc, and IGF-IR*β*, has the same molecular weight as the target protein Shc and this kit allows the immunoglobulin to remain adherent to Aminolink Plus Gel following elution. The procedures were done according to the manufacturer's protocol [9]. Briefly, 200 *μ*g of ER*α* rabbit polyclonal antibody (Santa Cruz) were coupled to 50 *μ*L of 50% of Aminolink Plus Gel Slurry in coupling buffer overnight at 4°C. The coupled gel and antibody complex was incubated with 300 *μ*g of the total protein harvested from the cells treated with E_2_ at 0, 10, and 60 min same as described in western blotting procedures in binding buffer overnight at 4°C. The gel was washed three times with washing buffer. Only the antigens (Shc and IGF-IR) in the antigen-antibody complexes were eluted by the elution buffer (all buffers were supplied in the kit) and stored for western blot analysis. 

### 2.7. Statistics

The experiments for RT-PCR of IGF-I mRNA expression, immunoprecipitation, and western blot analysis were repeated at least three times independently. The data obtained from RT-PCR were expressed as mean ± SEM, and the two-tailed Student's *t*-test was used to compare statistical significance between different groups and between various time points. Most data obtained from immunoprecipitation and western blot analyses were not normally distributed, hence, nonparametric statistical methods and Mann-Whitney tests [23] were used to determine statistically significant differences between silenced and nonsilenced IGF-IR groups at various time points after E_2_ treatment in UtLM cells with respect to ratio of band intensity of phosphorylated/total protein (mean ± SEM) of IGF-IR, Shc, MAPKp44/42, and ER*α*ser118 expression. The statistical significance was defined as one-sided *P* < 0.05. 

 For the RT^2^ Profiler PCR Array data, the receptor tyrosine kinase IGF-IR, MAPK signaling pathway, and fibrosis-related genes were chosen to be analyzed. The fold changes in response to E_2_ treatment were calculated separately for silenced and non-silenced IGF-IR groups (<−2 or >2 for either groups) and examined with scatter plots.

## 3. Results

### 3.1. Differential Expression of IGF-IR/MAPK-Related Genes Mediated by E_2_ with and without IGF-IR Gene Knockdown in UtLM Cells

**Figure 1:**
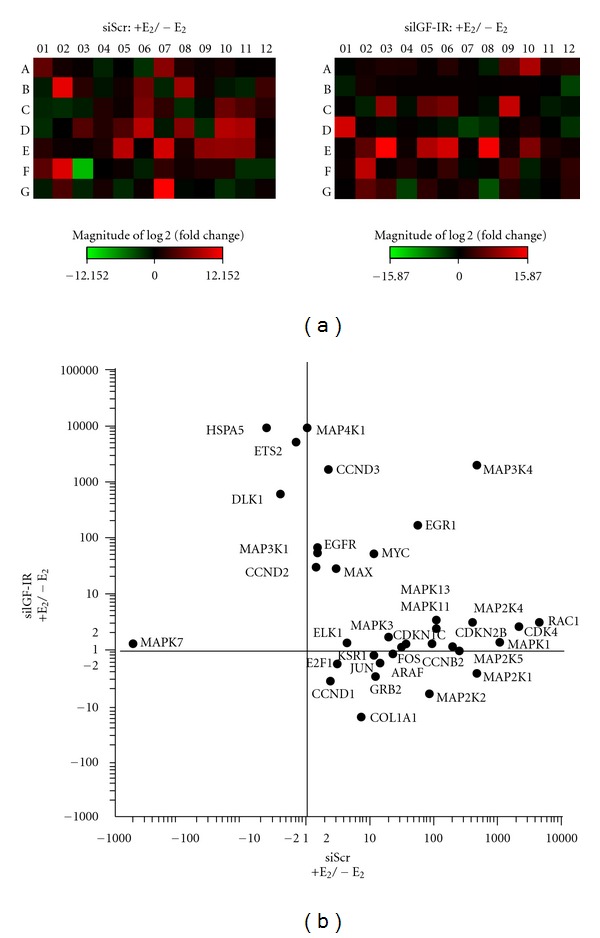
Differential MAPK pathway-related gene expression in UtLM cells mediated by 17*β*-estradiol (E_2_) in the presence of scrambled siRNA (siScr) or IGF-IR silencing (siIGF-IR). (a) Heat maps of real-time RT^2^ Profiler PCR Array of MAPK-related genes. The red areas represent the genes that are upregulated, and the green areas represent the genes that are downregulated by E_2_ treatment. (b) Plot of fold changes of MAPK-related genes in response to E_2_ treatment in UtLM cells with siScr or siIGF-IR.

In order to explore signaling intermediates of the IGF-IR/MAPK cascade and related genes regulated by E_2_ and find new genomic mediators of fibroid growth and development, we performed genomic arrays to determine MAPK-related gene profiles mediated by E_2_ with and without IGF-IR gene knockdown. We found 35 genes related to the IGF-IR/MAPK signaling pathway and fibrosis that were differentially expressed between the groups with or without E_2_ treatment at 24 h with a functional IGF-IR (scrambled siRNA; siScr), compared to those groups under siIGF-IR conditions (Table 1, Figure 1) within a total of 62 differentially expressed MAPK-related genes (see supplementary material available online at doi:10.1155/2012/204236, Table 1). The PCR array showed that E_2_ exposure in UtLM cells with a functional IGF-IR (siScr) resulted in >2-fold upregulation of 27 genes and <−2-fold downregulation of 3 genes involved in the IGF-IR/MAPK signaling cascade at 24 h as shown in a heat map (Figure 1(a)) and by fold changes (Figure 1(b)); the other 5 genes were unchanged (<2-fold and >−2-fold). Those genes upregulated >2-fold in the presence of an intact IGF-IR included several D-type cyclins and other cyclin-dependent kinases involved in cell cycle progression, growth factor receptor-bound protein (GRB2), and ARAF associated with IGF-IR signaling through MAPK, MAPKs, and MAPK kinases involved in proliferation, differentiation, and survival. Collagen type I alpha I (COL1A1) which is involved in collagen synthesis and fibrosis, a prominent feature of fibroids, and transcriptional factors ELK1, E2F1, and EGR1 were upregulated as well. Also, RAC1, a Rho GTPase, involved in the regulation of several cellular processes and often activated following stimulation of RTKs [24], such as IGF-IR was increased.

The upregulated genes induced by E_2_ in the presence of an intact IGF-IR were mostly abrogated, and the 5 unchanged genes observed in the presence of a functional IGF-IR were upregulated after IGF-IR was silenced in UtLM cells. The E_2_ treatment with siIGF-IR resulted in differential expression of >2-fold upregulation of additional genes such as epidermal growth factor receptor (EGFR) involved in cell growth and survival, cyclin D2 (CCND2) and D3 (CCND3) involved in cell cycle progression, v-Ets erythroblastosis virus E26 oncogene homolog 2 (ETS2), a transcriptional factor involved in cell development and tumorigenesis, delta-like 1 homolog (DLK1) involved in fibrosis, and the glucose-regulated protein 78 kDa (HSPA5) associated with monitoring protein transport (Table 1, Figures 1(a) and 1(b)). Alternatively, COL1A1 and GRB2, typically increased in the presence of a functional IGF-IR, showed decreased expression with silenced IGF-IR. However, the transcriptional factor EGR1 showed further upregulation when IGF-IR was silenced.

The differences in gene expression in response to E_2_ treatment with and without IGF-IR silencing indicate the important role of IGF-IR and its signaling molecules in E_2_-mediated activation of MAPK and MAPK-related pathways in UtLM cells.

### 3.2. 17*β*-Estradiol Upregulates IGF-I Gene and Protein Expression in UtLM Cells

**Figure 2:**
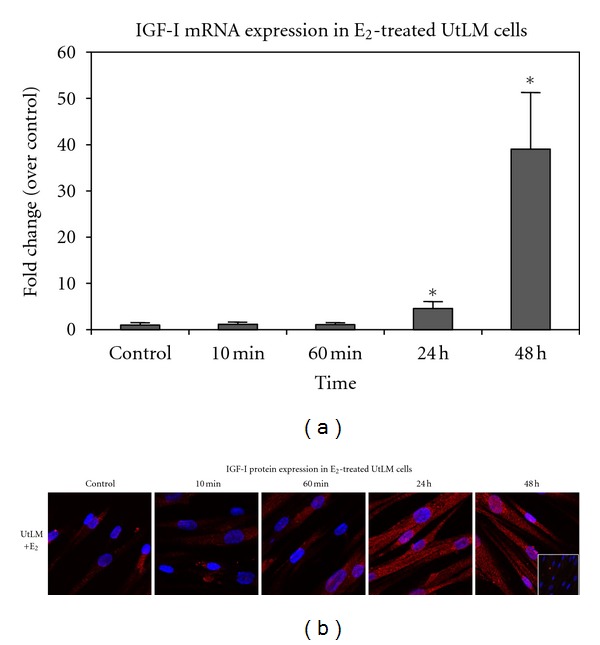
IGF-I mRNA and IGF-I peptide expression levels induced by 17*β*-estradiol (E_2_) in UtLM cells. (a) IGF-I gene expression in UtLM cells following E_2_ treatment at 0 (control), 10 and 60 min, and 24 h and 48 h. (b) IGF-I protein expression in UtLM cells following E_2_ exposure at 0 (control), 10 and 60 min, and 24 h and 48 h. Inset: Negative control with normal mouse IgG. Representative of mean ± SEM from three independent experiments. *P ≤ 0.05 versus control.

To determine if the regulatory effects of E_2_ on MAPK-related gene expression could also occur through the genomic action of E_2_ on the expression of one of its early response genes and a MAPK and IGF-IR activating peptide, IGF-I, we assessed IGF-I mRNA and protein levels in UtLM cells. We performed real-time RT PCR at 0, 10, 60 min, 24 h, and 48 h after E_2_ treatment and found that E_2_ at 10^−8^ M induced a time-dependent increase in IGF-I gene expression. A prolonged response started at 24 h and reached maximum levels by 48 h in UtLM cells (*P* < 0.05, Figure 2(a)). 

Confocal microscopy of immunofluorescence staining for IGF-I protein in UtLM cells further revealed that IGF-I peptide expression was increased by E_2_ and mostly localized in the cytoplasm, but appeared to translocate to the nucleus at 24 h and 48 h (Figure 2(b)). Interestingly, to date, IGF-I protein expression has only been reported to occur in the cytoplasm of uterine leiomyoma cells, although there has been one report of its localization in the nucleus of kidney cells [25].

### 3.3. Increased Phosphorylation of IGF-IR*β* by E_2_ Leads to MAPKp44/42 Activation and ER*α*  Phosphorylation at serine118 in UtLM Cells

**Figure 3:**
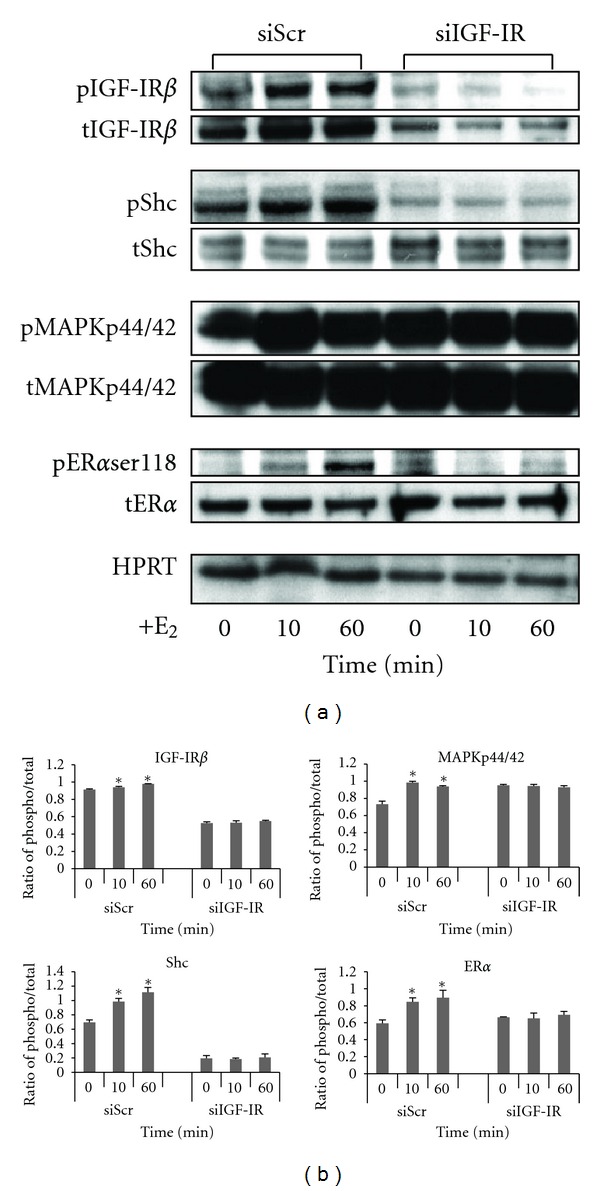
Differential expression of phosphorylated (p)IGF-IR, pMAPKp44/42, and pER*α*ser118 in UtLM cells with scrambled siRNA (siScr) or IGF-IR silencing (siIGF-IR) followed by 17*β*-estradiol (E_2_) treatment. (a) Western blot of IGF-IR/ER*α* pathway proteins in UtLM cells. (b) Comparison of ratio of densitometric band intensities of phosphorylated (phospho)/total proteins in UtLM cells with siScr or siIGF-IR followed by E_2_ treatment. Bars represent mean ± SEM of three independent experiments. *P < 0.05 versus 0 min.

**Figure 4:**
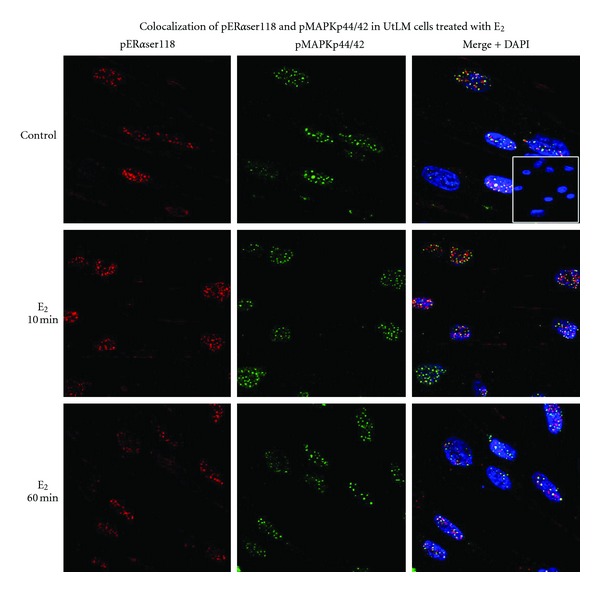
Increased colocalization of phosphorylated (p)MAPKp44/42 and pER*α*ser118 in UtLM cells exposed to 17*β*-estradiol (E_2_). Localization of pER*α*ser118 expression (red), pMAPKp44/42 (green) and colocalization of both (yellow) in UtLM cells following E_2_ treatment. The staining was primarily localized to the nucleus. Inset: Negative control with normal mouse and rabbit IgG. Representative of three independent experiments.

To determine the rapid nongenomic actions of E_2_, we treated UtLM cells with a functional IGF-IR with E_2_ at 10^−8^ M for 0, 10, and 60 min and measured phosphorylated IGF-IR*β*, Shc, and MAPKp44/42 using western blot analysis. We found that there was a quick phosphorylation of IGF-R*β*, Shc, and MAPKp44/42 at 10 min until 60 min in UtLM cells when treated with E_2_ (Figure 3). It has been previously shown that estrogen treatment can cause an increase in MAPKp44/42 activation and phosphorylation of ER*α* at serine118 site in estrogen-responsive breast cancer cell lines [26]. It has also been shown that the IGF-IR downstream protein Shc can act as a transporter for the ER leading to activation of the MAPK signaling cascade [27]. Additionally, in earlier studies, we found that fibroid tissue samples from women in the proliferative phase expressed more phosphorylated ER*α*ser118 and had more nuclear colocalization and immunoprecipitation of phospho-MAPKp44/42 and ER*α*ser118 compared to patient-matched myometrial controls [22]. We therefore examined whether administration of estrogen could produce similar effects in human uterine leiomyoma cells *in vitro*. As shown in Figures 3(a) and 3(b), the phosphorylation level of ER*α* at serine 118 rapidly increased at 10 min and continued until 60 min in UtLM cells following E_2_ treatment. These effects were not present with siIGF-IR. Confocal microscopy further revealed that there was increased colocalization of phosphorylated ER*α*ser118 and MAPKp44/42 in E_2_ treated UtLM cells at 10 min (Figure 4). 

### 3.4. IGF-IR Is Required to Modulate the ER*α* and MAPK Interaction in UtLM Cells Exposed to E_2_

**Figure 5:**
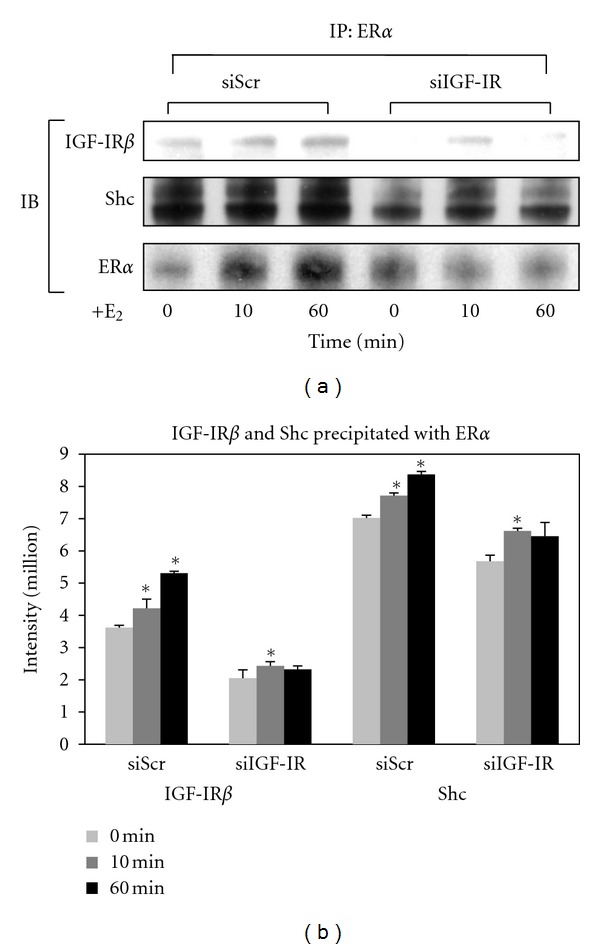
Increased immunoprecipitation of IGF-IR and Shc with ER*α* in cells exposed to 17*β*-estradiol (E_2_). (a) Interactions between IGF-IR*β* and Shc with ER*α* in UtLM cells were determined by immunoprecipitation (IP). (b) Comparison of densitometric band intensity of immunoblots (IB) in UtLM cells with siScr or siIGF-IR followed by E_2_ treatment. Representative of three independent experiments. Bars represent mean intensities ± SEM. *P < 0.05 versus 0 min.

Immunoprecipitation studies (Figure 5) further revealed that ER*α* is associated with both IGF-IR*β* and Shc proteins following E_2_ treatment, and the amounts of both proteins were decreased when the IGF-IR gene was knocked down, which indicates that there is an association between IGF-IR and ER*α* and between Shc and ER*α* in UtLM cells exposed to E_2_.

## 4. Discussion

The collective view of extranuclear ER*α* signaling suggests that its transduction pathways and its interaction with IGF-IR/MAPK pathways may connect the nongenomic actions of estrogen to genomic responses, since many of them interact and regulate the phosphorylation status and activities of multiple transcription factors, which affect gene expression [14, 27–30]. Therefore, in this study we first investigated the gene profile involved in the MAPK pathway to explore the effects of E_2_ treatment on IGF-IR/MAPK pathway related gene expression using siIGF-IR and real-time PCR array technology. As shown in Table 1 and Figure 1, UtLM cells treated with E_2_ in the presence of an intact IGF-IR had a high induction of 27 genes including genes encoding Cyclins, Cyclin Kinases, MAPKs, MAPK kinases, and transcription factors all involved in the cell proliferation, differentiation and survival. However, by silencing the IGF-IR the effects induced by E_2_ were diminished in UtLM cells and further indicated that E_2_-mediated MAPK pathway activation requires the presence of the IGF-IR in UtLM cells. 

A cascade of MAPKs can be induced by a variety of signaling molecules [31, 32]. Transduction of the signals is achieved by a sequential series of phosphorylation reactions, wherein each downstream kinase serves as a substrate for the upstream activator. For example, in the mitogenic extracellular signal regulated kinase (ERK1/2) cascade, the two related mammalian MAPKs, ERK1 and ERK2 (p44mapk and p42mapk), are phosphorylated by MAP kinase/ERK kinase (MEK), which is activated primarily by the protein kinase Raf-1 after having been recruited to the plasma membrane by Ras [33, 34]. In our previous studies, the MAPKp44/42 cascade, preferentially regulating cell growth and differentiation, was upregulated in leiomyomas [9, 22]. The MAPK-related gene profiling RT-PCR array applied in this study further proves that the expression of genes involved in MAPK pathway, such as MAPK1, MAPK3, and GRB2, and the genes encoding transcriptional factors, ELK1, E2F1, MYC, and MAX are mediated by E_2_, and their expression levels are elevated when UtLM cells are exposed to E_2_ in the presence of a functional IGF-IR.

In the highly conserved cyclin family, whose members are characterized by a dramatic periodicity in protein abundance throughout the cell cycle, cyclin D1 can complex with and function as a regulatory subunit of CDK4 or CDK6, which is required for cell cycle G1/S progression [35]. In this study, cyclin D1 and CDK4 expression levels were increased, which further indicates that E_2_ not only upregulates the MAPK/ERK1/2 pathway, but can also induce cyclin family proteins leading to cell cycle progression from G1 to S phase, thereby increasing cell proliferation. It was interesting to note that some genes encoding cyclin kinase inhibitors, such as CDKN2B, were also increased following E_2_ exposure and may have been increased to counterbalance the extremely high expression of CDK4. These findings are consistent with the concept of different cyclins and their kinases exhibiting distinct expression and degradation patterns which contribute to the temporal coordination of each mitotic event [35].

The abrogation of upregulation of genes by E_2_ in the presence of siIGF-IR in UtLM cells further strengthened our hypothesis that IGF-IR plays an important role in the crosstalk between the estrogen/ER*α* and IGF-I/MAPKp44/42 pathways. However, other genes were differentially expressed by E_2_ treatment in UtLM cells with and without siIGF-IR, which indicates that with silencing of the IGF-IR, other mechanisms compensated in response to E_2_ exposure, resulting in differential gene expression. The increased EGFR, EGR1, CCND2, and CCND3 gene expression pattern with siIGF-IR suggests that E_2_ treatment promotes alternative pathways for growth and survival when IGF-IR levels are decreased in UtLM cells. In other genes, such as DLK-1, involved in fibrosis [36], the expression level was increased, and COL1A1, a gene involved in collagen synthesis and fibrosis, which is typically increased in the presence of a functional IGF-IR [37, 38], was decreased after siIGF-IR further indicating that IGF-IR may play an important role in fibrosis in uterine leiomyomas.

We next investigated whether E_2_ upregulates IGF-I and IGF-IR, and their target proteins in leiomyoma cells, and what mechanisms are involved in the interaction between E_2_/ER*α* and IGF-I/IGF-IR pathways. We found that IGF-I gene and protein expression levels increased during the course of E_2_ exposure with a peak fold-change at 48 hours in UtLM cells; this prolonged response indicates a possible mechanism of IGF-I gene expression mediated by E_2_ at a genomic level. We also found that phosphorylated ER*α*ser118, IGF-IR*β*, and their target protein MAPKp44/42 were all increased within minutes after E_2_ treatment. The rapid activation of IGF-IR and its target downstream proteins indicates that the interaction between E_2_ and IGF-IR is mediated by nongenotropic signaling, that is, by kinase-initiated events that do not involve estrogen receptor binding to canonical steroid response elements on DNA [18, 28]. Furthermore, increased colocalization of phospho-MAPKp44/42 and ER*α*ser118 occurred in UtLM cells 10 minutes after E_2_ treatment. These results are consistent with the findings that several mechanisms are associated with E_2_ exposure, including rapid activation of IGF-IR and MAP kinase, a nongenomic process observed in estrogen-responsive breast cancer cell lines [39], which could lead to ER*α* activation at serine 118 [14, 28]. 

The molecules involved in the nongenomic signaling process have been identified. More recently, it has been shown that a pool of ERs resides in or is associated with the plasma membrane. These ERs utilize the membrane IGF-IR to rapidly signal through various kinase cascades that influence both transcriptional and nontranscriptional actions of estrogen [28]. In this study, UtLM cells treated with E_2_, showed upregulation of RAC1, a Rho GTPase involved in the regulation of several cellular processes that is often activated following stimulation of RTKs [24]. This gene expression of RAC1 was decreased upon IGF-IR silencing. These data indicate that E_2_ can directly, or through the involvement of the ER*α*, activate IGF-IR and MAPK signaling. The IGF-IR may serve as an anchor for the plasma membrane-associated ER*α*. Estradiol causes rapid phosphorylation of IGF-IR and Shc. It has been reported that activated Shc, after binding to ER*α*, serves as a transporter, which carries ER*α* to Shc-binding sites on the activated IGF-I receptors [39], which subsequently signals to MAPKs and other pathways. Our immunoprecipitation results also show that these three proteins, ER*α*, IGF-IR, and Shc, are associated with each other when UtLM cells are treated with E_2_. Therefore, we proposed that IGF-IR should be a key mediator in this interaction, and applied siIGF-IR methodology to knockdown the IGF-IR gene to block the E_2_ effect on the interaction of these two pathways through their respective receptors. We found that siIGF-IR decreased the phosphorylation of IGF-IR*β* and the activation of MAPKp44/42 induced by E_2_. At same time, ER*α* phosphorylation at the serine118 site was also attenuated. These findings indicate that IGF-IR*β* activation is required in the rapid nongenomic response of ER*α* following E_2_ exposure in UtLM cells. Silencing of IGF-IR abrogated the ER*α* activity at the serine118 site induced by E_2_ confirming a potential relationship between membrane-related signals and intracellular ER*α*, in agreement with the findings that E_2_ binds to cell membrane-associated ER*α*, which physically associates with the adaptor protein Shc through IGF-IR activation and induces its phosphorylation [39]. In turn, Shc binds GRB2 and Sos, which also results in the rapid activation of MAP kinase, and we have shown an association between IGF-IR and Grb2 and MAP kinase activation in fibroid tissue samples taken from women in the proliferative phase of the menstrual cycle [9, 40].

**Figure 6:**
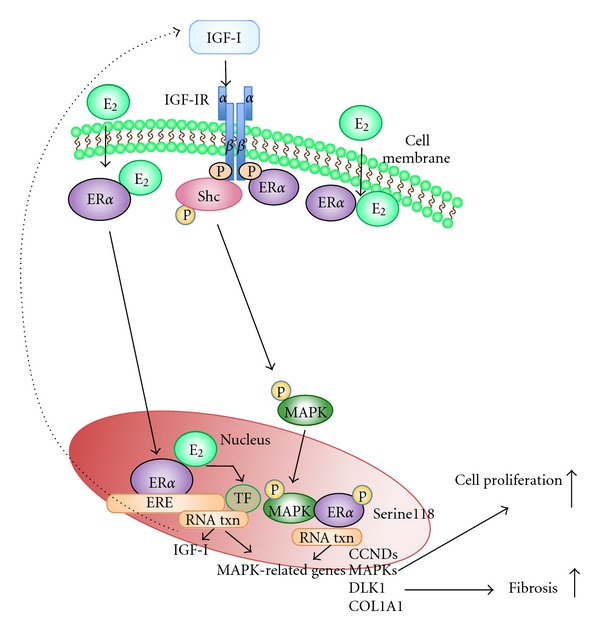
Schematic illustration of genomic and nongenomic actions of ER*α* on target gene transcription. Genomic actions involve the translocation of cytoplasmic E_2_-ER*α* complexes to the nucleus which can then bind directly to estrogen response elements (EREs) in target gene promoters or nuclear E_2_-ER*α* complexes. These complexes are tethered through protein-protein interactions to a transcription factor complex (TF) that contacts the target gene promoter to induce transcription of IGF-I and MAPK related genes. Nongenomically, E_2_ can bind to membrane associated ER*α* which then binds to the adaptor protein, Src collagen homologue (Shc) to form a protein complex consisting of ER*α* and Shc and/or ER*α* and IGF-IR. E_2_ signals through the IGF-IR and activates MAPKp44/42, which can then phosphorylate ER*α* at the serine118 site to initiate transcription (txn) of MAPK related genes. CCNDs = Cyclin Ds; MAPKs = mitogen-activated protein kinases; DLK1 = delta-like 1 homolog; COL1A1 = collagen type I alpha 1.

Therefore, the possible convergence of distinct ER*α*-mediated genomic and/or nongenomic actions at multiple response elements provides an extremely fine control system in the regulation of target gene transcript [14] leading to the alternation of gene expression profiles found in this study. In conclusion, the results obtained in this study indicate that the two growth regulatory pathways, E_2_/ER*α* and IGF-I/IGF-IR, are tightly linked in UtLM cells. The E_2_ effects can occur through both genomic and nongenomic events, which involve IGF-IR activation of MAP kinase cascades mediated by the association between ER*α*ser118 and MAPKp44/42 (Figure 6). The observations that: (1) IGF-IR is required for the interaction and the differential expression of MAPK pathway-related genes mediated by E_2_; (2) IGF-I gene expression is responsive to E_2_; (3) the activation of alternative pathways induced by E_2_ when IGF-IR is silenced enhances our understanding of IGF-I/IGF-IR and E_2_/ER*α* interactions and may suggest a multipronged or cocktail approach to fibroid treatment. Considering that the pure antiestrogen or anti-IGF-IR agents may only be partially effective in antagonizing E_2_-induced IGF-I/MAPK pathway activation and because other alternative pathways (EGFR) could compensate, it suggests that inhibitors of small downstream molecules, such as Src and ERKs, or transcription factors may better block these effects and could possibly serve as noninvasive adjuvant therapies for fibroids. 

## Supplementary Material

The RT2 Profiler PCR Array was used to analyze MAPK pathway-related gene expression in human uterine leiomyoma (UtLM) cells mediated by E_2_ in the presence of scrambled siRNA (siScr) or IGF-IR silencing (siIGF-IR). There were 62 genes related to the MAPK signaling differentially expressed between the UtLM cells with a functional IGF-IR (siScr) compared to UtLM cells under siIGF-IR conditions in the presence or absence of E_2_ treatment at 24 h(>2-fold, upregulation and <−2-fold, downregulation).

## Figures and Tables

**Table 1:** Differential MAPK-related gene expression in uterine leiomyoma (UtLM) cells with scrambled siRNA (siScr) or IFG-IR silencing (siIGF-IR) followed by E_2_ treatment.

Heat map	Symbol	Accession	Description	siScr	silGF-IR
position		number		+E_2_/−E_2_	+E_2_/−E_2_
A01	ARAF	NM_001654	V-raf murine sarcoma viral oncogene homolog, transduction of mitogenic signal to nucleus	23.3	−1.2
A07	CCNB2	NM_004701	Cyclin B2, related to transforming growth factor beta-mediated cell cycle control	94.0	1.3
A08	CCND1	NM_053056	Cyclin Dl, cell cycle regulation, Gl-S transition	2.4	−3.7
A09	CCND2	NM_001759	Cyclin D2, cell cycle regulation, Gl-S transition	1.4	29.7
A10	CCND3	NM_001760	Cyclin D3, cell cycle regulation, Gl-S transition	2.3	1652.0
B02	CDK4	NM_000075	Cyclin-dependent kinase 4, A subunit of protein kinases complex in cell cycle G1 phase	2148.2	2.5
B06	CDKN1C	NM_000076	Cyclin-dependent kinase inhibitor 1C (p57, Kip2)	37.6	1.3
B08	CDKN2B	NM_004936	Cyclin-dependent kinase inhibitor 2B (p15, inhibits CDK4)	198.8	1.1
B12	COL1A1	NM_000088	Collagen, type I, alpha 1	7.3	−15.8
C03	DLK1	NM_003836	Delta-like 1 homolog (*Drosophila*), contains EGF-like repeat, related to fibrosis	−2.5	604.7
C04	E2F1	NM_005225	E2F transcription factor 1	3.1	−1.9
C05	EGFR	NM_005228	Epidermal growth factor receptor	1.5	67.2
C06	EGR1	NM_001964	Early growth response 1, a zinc finger protein, and nuclear transcriptional regulator	55.5	166.6
C07	ELK1	NM_005229	Transcription factor, a nuclear target of ras-raf-MAPK signaling cascade	4.4	1.3
C09	ETS2	NM_005239	V-Ets erythroblastosis virus E26 oncogene homolog 2 (avian), a transcriptional factor	−1.4	>5000
C10	FOS	NM_005252	Leucine-zip-protein, dimerizes with Jun, involved in AP-1 complex	32.8	1.1
C11	GRB2	NM_002086	Growth factor receptor-bound protein 2	12.4	−3.0
D01	HSPA5	NM_005347	Heat shock 70 kDa protein 5 (glucose-regulated protein, 78 kDa), related to protein transport in cells	−4.0	>5000
D03	JUN	NM_002228	Jun oncogene, interacts with target DNA sequence to regulate gene expression	14.7	−1.7
D05	KSR1	NM_014238	A scaffold protein connecting MEK to RAF	12.1	−1.3
D06	MAP2K1	NM_002755	Mitogen-activated protein kinase kinase 1	470.9	−2.7
D08	MAP2K2	NM_030662	A MAP kinase kinase, activates MAPK1/ ERK2 cascade	84.2	−6.2
D10	MAP2K4	NM_003010	A MAP kinase kinase, activates MAPK8/JUK cascade	411.0	3.0
D11	MAP2K5	NM_002757	A MAP kinase kinase, activates MAPK7/ERK5 cascade	253.7	−1.1
E02	MAP3K1	NM_005921	A MAP kinase kinase, activates ERK/JUK cascade	1.5	53.1
E05	MAP3K4	NM_005922	A MAP kinase kinase, activates JUK/MAPK cascade	475.8	1951.0
E06	MAP4K1	NM_007181	A MAP kinase kinase, acts upstream of JUN-N terminal pathway	1.0	>5000
E07	MAPK1	NM_002745	Mitogen-activated protein kinase, encoding of MAPKp42, activates Elk-1	1101.7	1.3
E09	MAPK11	NM_002751	A MAP kinase kinase related to p38	111.1	2.4
E11	MAPK13	NM_002754	A MAP kinase kinase related to p38	107.8	3.2
F01	MAPK3	NM_002746	Mitogen-activated protein kinase 3, encoding of MAPKp44, activates Elk-1	19.9	1.6
F03	MAPK7	NM_002749	Mitogen-activated protein kinase 7, encoding of ERK4/5	−495.9	1.3
F09	MAX	NM_002382	MYC-associated factor X, a transcription factor	3.0	28.1
G02	MYC	NM_002467	V-myc myelocytomatosis viral oncogene homolog (avian), a transcription factor	11.4	51.3
G07	RAC1	NM_006908	Ras-related C3 botulinum toxin substratel (Rho family), GTPase of Ras family of small GTP-binding proteins	4552.5	3.1
