# Supplementary material for: Estrogen Regulates MAPK-Related Genes through Genomic and Nongenomic Interactions between IGF-I Receptor Tyrosine Kinase and Estrogen Receptor-Alpha Signaling Pathways in Human Uterine Leiomyoma Cells
Source: J Signal Transduct. 2012 Oct 9;2012:204236. doi: 10.1155/2012/204236 (PMC3474284; doi:10.1155/2012/204236)
Supplement: Supplementary file 1 — The RT2 Profiler PCR Array was used to analyze MAPK pathway-related gene expression in human uterine leiomyoma (UtLM) cells mediated by E2 in the presence of scrambled siRNA (siScr) or IGF-IR silencing (siIGF-IR). There were 62 genes related to the MAPK signaling differentially expressed between the UtLM cells with a functional IGF-IR (siScr) compared to UtLM cells under siIGF-IR conditions in the presence or absence of E2 treatment at 24 h(>2-fold, upregulation and <−2-fold, downregulation). [file 204236.f1.pdf]

Supplemental Table. MAPK related gene expression in uterine leiomyoma (UtLM) cells with scrambled siRNA (siScr) or IGF-IR silencing (siIGF-IR) followed by E_2 treatment

Heatmap Position	Symbol	Accession Number	Description	siScr $+E_2/-E_2$	siIGF-IR $+E_2/-E_2$
A01	ARAF	NM_001654	V-raf murine sarcoma viral oncogene homolog, transduction of mitognic signal to nucleus	23.3	-1.2
A04	CCNA1	NM_003914	Activating transcription factor 2, regulatos of CDK kinases in the control of the germline meiotic cell cycle	-3.1	3.2
A06	CCNB1	NM_031966	Cyclin B1, complexes with p34(cdc2) to form the maturation-promoting factor (MPF).	-5.0	4.6
A07	CCNB2	NM_004701	Cyclin B2, related to transforming growth factor beta-mediated cell cycle control	94.0	1.3
A08	CCND1	NM_053056	Cyclin D1, cell cycle regulation, G1-S transition	2.4	-3.7
A09	CCND2	NM_001759	Cyclin D2, cell cycle regulation, G1-S transition	1.4	29.7
A10	CCND3	NM_001760	Cyclin D3, cell cycle regulation, G1-S transition	2.3	1652.0
B01	CDK2	NM_001798	Cyclin-dependent kinase 2, regulated by complex of cyclin A or E, CDK inhibitor p21Cip1 and p27Kip1	-2.3	-3.3
B02	CDK4	NM_000075	Cyclin-dependent kinase 4, A subunit of protein kinases complex in cell cycle G1 phase	2148.2	2.5
B03	CDK6	NM_001259	Cyclin-dependent kinase 6, in mid-G1 phase controlled by D-type cyclins and INK4 family of CDK inhibitors	3.7	1.5
B06	CDKN1C	NM_000076	Cyclin-dependent kinase inhibitor 1C (p57, Kip2)	37.6	1.3
B07	CDKN2A	NM_000077	Cyclin-dependent kinase inhibitor 2A (melanoma, p16, inhibits CDK4)	-3.4	1.3
B08	CDKN2B	NM_004936	Cyclin-dependent kinase inhibitor 2B (p15, inhibits CDK4)	198.8	1.1
B10	CDKN2D	NM_001800	Cyclin-dependent kinase inhibitor 2D (p19, inhibits CDK4)	-2.1	1.3
B11	CHUK	NM_001278	Conserved helix-loop-helix ubiquitous kinase	-3.1	1.1
B12	COL1A1	NM_000088	Collagen, type I, alpha 1	7.3	-15.8
C01	CREB1	NM_004379	CAMP responsive element binding protein 1, a transcription factor	-3.2	1.3
C02	CREBBP	NM_004380	CREB binding protein, a DNA binding protein of transcription factor of leucine zipper family	-4.1	-4.1
C03	DLK1	NM_003836	Delta-like 1 homolog (Drosophila), contains EGF-like repeat, related to fibrosis	-2.5	604.7
C04	E2F1	NM_005225	E2F transcription factor 1	3.1	-1.9
C05	EGFR	NM_005228	Epidermal growth factor receptor	1.5	67.2
C06	EGR1	NM_001964	Early growth response 1, a zinc finger protein, and nuclear transcriptional regulator	55.5	166.6
C07	ELK1	NM_005229	Transcription factor, a nuclear target of ras-raf-MAPK signaling cascade	4.4	1.3
C08	ETS1	NM_005238	a ETS transcriptions factor, involved in stem cell development, cell death, and tumorigenesis	-4.3	-1.9
C09	ETS2	NM_005239	V-Ets erythroblastosis virus E26 oncogene homolog 2 (avian), a transcriptional factor	-1.4	>5000
C10	FOS	NM_005252	Leucine-zip-protein, dimerizes with Jun, involved in AP-1 complex	32.8	1.1
C11	GRB2	NM_002086	Growth factor receptor-bound protein 2	12.4	-3.0
C12	HRAS	NM_005343	V-Ha-ras Harvey rat sarcoma viral oncogene homolog, a GTPase activates proteins such as c-Raf and PI 3-kinase	3.6	-1.9
D01	HSPA5	NM_005347	Heat shock 70kDa protein 5 (glucose-regulated protein, 78kDa), related to protein transport in cells	-4.0	>5000
D03	JUN	NM_002228	Jun oncogene, interacts with target DNA sequence to regulate gene expression	14.7	-1.7
D04	KRAS	NM_004985	V-Ki-ras2 Kirsten rat sarcoma viral oncogene homolog, activates proteins such as c-Raf and PI 3-kinase	3.2	1.3
D05	KSR1	NM_014238	A scaffold protein connecting MEK to RAF	12.1	-1.3
D06	MAP2K1	NM_002755	Mitogen-activated protein kinase kinase 1	470.9	-2.7
D08	MAP2K2	NM_030662	A MAP kinase kinase, activates MAPK1/ ERK2 cascade	84.2	-6.2
D09	MAP2K3	NM_002756	A MAP kinase kinase, activates MAPK14/p38-MAPK .	-4.3	1.3
D10	MAP2K4	NM_003010	A MAP kinase kinase, activates MAPK8/JUK cascade	411.0	3.0
D11	MAP2K5	NM_002757	A MAP kinase kinase, activates MAPK7/ERK5 cascade	253.7	-1.1
E02	MAP3K1	NM_005921	A MAP kinase kinase, activates ERK/JUK cascade	1.5	53.1
E04	MAP3K3	NM_002401	A MAP kinase kinase, regulates the stress-activated protein kinase (SAPK)	2.3	1.8
E05	MAP3K4	NM_005922	A MAP kinase kinase, activates JUK/MAPK cascade	475.8	1951.0
E06	MAP4K1	NM_007181	A MAP kinase kinase, acts upstream of JUN-N terminal pathway	1.0	>5000
E07	MAPK1	NM_002745	Mitogen-activated protein kinase, encoding of MAPKp42, activates Elk-1	1101.7	1.3
E09	MAPK11	NM_002751	A MAP kinase kinase related to p38	111.1	2.4
E10	MAPK12	NM_002969	Mitogen-activated protein kinase 12, a signal transducer during differentiation of myoblasts to myotubes	136.2	226.0
E11	MAPK13	NM_002754	A MAP kinase kinase related to p38	107.8	3.2
F01	MAPK3	NM_002746	Mitogen-activated protein kinase 3, encoding of MAPKp44, activates Elk-1	19.9	1.6
F02	MAPK6	NM_002748	Mitogen-activated protein kinase 6 (p97), also known as extracellular signal-regulated kinases (ERKs)	1532.6	3717.2
F03	MAPK7	NM_002749	Mitogen-activated protein kinase 7, encoding of ERK4/5,	-495.9	1.3
F06	MAPK9	NM_002752	Mitogen-activated protein kinase 9, related to MAPK8, involved in UV induced apoptosis, and cell death pathway	-2.6	9.6
F07	MAPKAP	NM_004759	Mitogen-activated protein kinase-activated protein kinase 2	4.8	-1.3
F09	MAX	NM_002382	MYC associated factor X, a transcription factor	3.0	28.1
F10	MEF2C	NM_002397	Myocyte enhancer factor 2C, transcription activator binds to the MEF2 regulating cardiac muscle-specific genes	2.8	-3.7
F11	MKNK1	NM_003684	MAP kinase interacting serine/threonine kinase 1, in the response to environmental stress and cytokines	-4.3	1.5
F12	MOS	NM_005372	V-mos Moloney murine sarcoma viral oncogene homolog, Proto-oncogene serine/threonine-protein kinase	-4.3	7.5
G01	MST1	NM_020998	Macrophage stimulating 1 (hepatocyte growth factor-like)	-2.4	1.1
G02	MYC	NM_002467	V-myc myelocytomatosis viral oncogene homolog (avian), a transcription factor	11.4	51.3
G03	NFATC4	NM_004554	Nuclear factor of activated T-cells, cytoplasmic, calcineurin-dependent 4	-3.0	12.4
G04	NRAS	NM_002524	Neuroblastoma RAS viral (v-ras) oncogene homolog, encoded protein has intrinsic GTPase activity	2.1	-16.4
G05	PAK1	NM_002576	P21 protein (Cdc42/Rac)-activated kinase, link RhoGTPases to cytoskeleton reorganization and nuclear signaling	-3.7	6.9
G07	RAC1	NM_006908	Ras-related C3 botulinum toxin substrate 1 (rho), Ras-related GTPase of RAS family of small GTP-binding proteins	4552.5	3.1
G10	SFN	NM_006142	Stratifin, p53-regulated inhibitor of G2/M progression	-4.3	-1.9
H03	RPL13A	NM_012423	Ribosomal protein L13a	-2.2	1.1
